# Supplementary material for: Discovery of a weak topological insulating state and van Hove singularity in triclinic RhBi2
Source: Nat Commun. 2021 Mar 25;12:1855. doi: 10.1038/s41467-021-22136-w (PMC7994551; doi:10.1038/s41467-021-22136-w)
Supplement: Supplementary file 1 — Supplementary Information [file 41467_2021_22136_MOESM1_ESM.pdf]

Supplementary information: Discovery of a weak topological insulating state and van Hove singularity in triclinic $RhBi_2$

Kyungchan Lee,[1,2] Gunnar F. Lange,[3] Lin-Lin Wang,[1,2] Brinda Kuthanazhi,[1,2] Thaís V. Trevisan,[1,2] Na Hyun Jo,[1,2] Benjamin Schrunk,[1,2] Peter P. Orth,[1,2] Robert-Jan Slager,[3,4] Paul C. Canfield,[1,2] and Adam Kaminski[1,2]

[1] *Ames Laboratory, Ames, Iowa 50011, USA*

[2] *Department of Physics and Astronomy, Iowa State University, Ames, Iowa 50011, USA*

[3] *TCM Group, Cavendish Laboratory, University of Cambridge, Cambridge CB3 0HE, United Kingdom*

[4] *Department of Physics, Harvard University, Cambridge MA 02138, USA*

(Dated: February 19, 2021)

SUPPLEMENTARY NOTE 1: CRYSTAL GROWTH AND CHARACTERIZATION

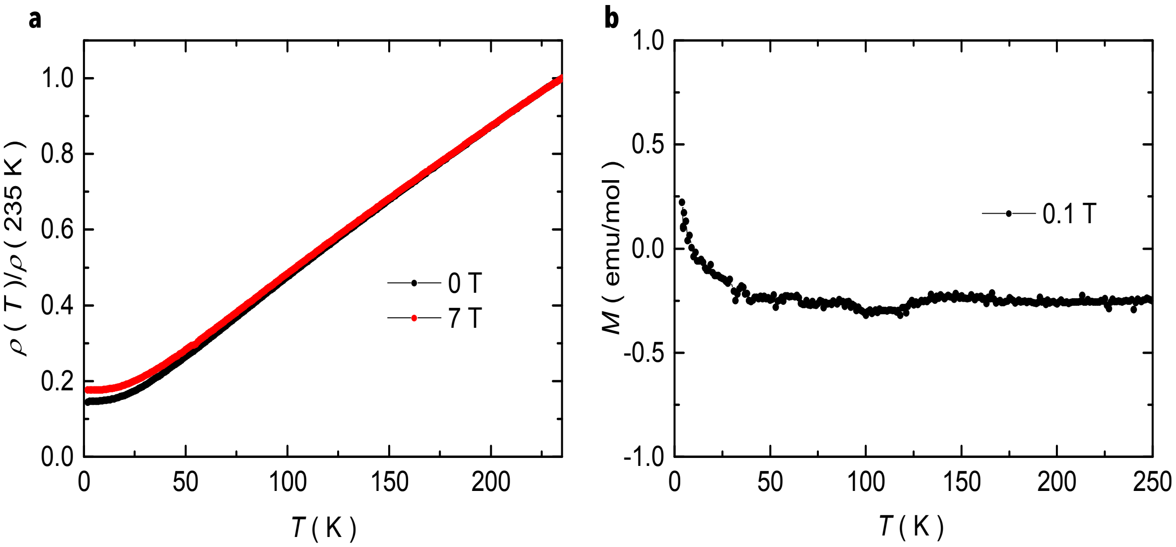

Supplementary Figure 1. Resistivity and magnetization data for $RhBi_2$. **a**, Resistivity data measured in zero field and 7 T. **b**, magnetization measured as a function of temperature in 0.1 T field.

Resistivity as a function of temperature was measured with current in the *bc*-plane, along one of the crystallographic axis. ρ(T) shows clear metallic behavior with no features that would suggest any sort of phase transition in the measured temperature range. With an applied field of 7 T, ρ(T) do not show any significance changes, except slightly higher resistance values at low temperatures. This very small magnetoresistance is not inconsistent with the relatively low RRR value ($\sim$ 6) of the sample. Magnetization as a function of temperature, M(T), was measured with field applied perpendicular to the *a*-axis, and shows nearly temperature independent behavior, with a small, negative value of M over most of the temperature range. This fundamentally diamagnetic response is consistent with a very small density of states at the Fermi energy resulting in a larger core diamagnetic component.

SUPPLEMENTARY NOTE 2: DETAILS OF THE EFFECTIVE MODEL

We aim to build an effective model for the observed surface state close to the $\overline{Z}$ point. As we know the form of the symmetries in the bulk, it would perhaps be most transparent to build a bulk model, which we then explicitly break to the relevant surface. In this section, we discuss this approach and explain why we instead opt to build a surface Hamiltonian directly. We also discuss the construction of this surface Hamiltonian.

A minimal bulk Hamiltonian requires four bands, due to the presence of doubly degenerate bulk Kramer's pairs. A general four-band model can be written in terms of 5 Γ matrices, and their corresponding commutators. However, we can restrict our model by imposing the bulk symmetries. The bulk has inversion and time-reversal symmetry. Combining these gives an operator $\mathcal{A} = \Theta\mathcal{P}$ which satisfies:

$$\mathcal{A}H(\mathbf{k})\mathcal{A}^{-1} = H(\mathbf{k}) \tag{1}$$

We can choose our Γ-matrices to commute with the $\mathcal{A}$ operator[1]. Requiring this gives that the commutators are all zero, so the effective model can be written using only the 5 Γ-matrices. We can represent our symmetry operations as[1]:

$$\mathcal{P} = \sigma_x \otimes I, \quad \Theta = i(I \otimes s_y)\mathcal{K} \tag{2}$$

Where $\mathcal{K}$ is complex conjugation. This gives the 5 Γ-matrices commuting with $\mathcal{A}$ and satisfying the Clifford algebra as:

$$\Gamma^{(1,2,3,4,5)} = \{\sigma_x \otimes I, \sigma_y \otimes I, \sigma_z \otimes s_x, \sigma_z \otimes s_y, \sigma_z \otimes s_z\} \tag{3}$$

Note that Γ^1 equals $\mathcal{P}$. We can then expand the Hamiltonian as:

$$H(\mathbf{k}) = \epsilon I_{4\times4} + M\Gamma^1 + \sum_{i=2}^{5} A_i \Gamma_i = \epsilon I_{4\times4} + M\Gamma^1 + S \tag{4}$$

Now we note that:

$$\Theta\Gamma^a\Theta^{-1} = \mathcal{P}\Gamma^a\mathcal{P}^{-1} = \begin{cases} 1 & \text{if } a = 1 \\ -1 & \text{if } a \neq 1 \end{cases} \tag{5}$$

From which it follows that ϵ and M are even in $\mathbf{k} \to -\mathbf{k}$, whereas the $\{A_i\}$ are odd. Furthermore, S anticommutes with Γ^1, which which it follows that:

$$\mathcal{P}H(k)\mathcal{P}^{-1} = \Gamma_1 H(k)\Gamma_1 = \epsilon I_{4\times4} + M\Gamma_1 - S \tag{6}$$

To get the surface states from this bulk Hamiltonian, we organize our Hamiltonian into a term H_0 which contains constants and k_x dependent terms, and a perturbation H_1 which does not contain any k_x dependence. As there is no preferred direction under the symmetry operations, H_0 contains all 5 Γ-matrices, and is therefore not block diagonal. We can then in principle solve H_0 on a finite slab geometry, which would give us four states, two of which are regular on the boundary. These can be interpreted as the edge states. From equation 6, it follows that the opposite parity states will be associated with an opposite sign for matrix S. Explicitly diagonalizing H_0 gives the eigenvectors:

$$v_1 = \begin{pmatrix} a \\ b \\ 1 \\ 0 \end{pmatrix}, v_2 = \begin{pmatrix} c \\ d \\ 0 \\ 1 \end{pmatrix}, v_3 = \begin{pmatrix} e \\ f \\ 1 \\ 0 \end{pmatrix}, v_4 = \begin{pmatrix} g \\ h \\ 0 \\ 1 \end{pmatrix} \tag{7}$$

Where the states (v_1, v_2) and (v_3, v_4) have pairwise the same eigenvalues, and the coefficients are complicated functions of the free parameters. These are not block diagonal as H_0 is not block diagonal. We could now in principle investigate which of these four states remain regular at the boundary, given some boundary conditions, and then project the perturbation H_1 in this basis. Note, however, that the resulting expression will be rather convoluted, with many free parameters. As we are only interested in the surface dispersion to compute the DOS, we choose to instead build a model for the surface directly.

The surface close to the TRIM $\overline{Z}$ exhibits time-reversal symmetry. Inversion symmetry is broken at the surface. However, there will be some residual effects of inversion symmetry, as otherwise H_0 should contain more terms, coming from the commutators of the Γ-matrices. Thus, inversion symmetry is broken down to some effective symmetry on the surface. The exact nature of this symmetry will depend on the orbital contents. Note in particular that if inversion acted purely as an effective C_2 symmetry, taking $(k_y, k_z) \to (-k_y, -k_z)$, then our expansion in equation 13 in the main text would be trivial. The exact nature of this effective symmetry can be elucidated by solving the full bulk Hamiltonian above. For our purposes, however, it suffices to ignore this residual symmetry, and only focus on the time-reversal symmetry. The residual inversion symmetry will only constrain the form of our effective Hamiltonian further, potentially resulting in less free parameters. Thus, we do not lose any physical solutions by only ignoring the residual inversion symmetry, though we may find an unphysical parameter combination. As we are only interested in the shape of the surface

dispersion, this is of no concern.

The time-reversal symmetry $\Theta = i\sigma_y \mathcal{K}$ acts in spin space on the surface by constraining:

$$H(\mathbf{k}) = (i\sigma_y)H^*(-\mathbf{k})(i\sigma_y)^{-1} \tag{8}$$

We can write a two-band model for the surface states as:

$$H(\mathbf{k}) = d(\mathbf{k}) \cdot \sigma \tag{9}$$

Hermiticity then requires that $d(\mathbf{k})$ should be real. Imposing time-reversal symmetry gives:

$$d_0(\mathbf{k}) = d_0(-\mathbf{k}), \quad d_1(\mathbf{k}) = -d_1(-\mathbf{k}), \quad d_2(\mathbf{k}) = -d_2(-\mathbf{k}), \quad d_3(\mathbf{k}) = -d_3(-\mathbf{k}) \tag{10}$$

With energy given by:

$$E(k) = d_0 \pm \sqrt{d_1^2 + d_2^2 + d_3^2} \tag{11}$$

Expanding to second order then gives:

$$H_2(k) = (E_0 + A_1 k_y^2 + A_2 k_z^2 + A_3 k_y k_z)I + (B_1 k_y + C_1 k_z)\sigma_x + (B_2 k_y + C_2 k_z)\sigma_y + (B_3 k_y + C_3 k_z)\sigma_z \tag{12}$$

With energy

$$E_{2\pm}(k) = E_0 + A_1 k_y^2 + A_2 k_z^2 + A_3 k_y k_z \pm \sqrt{\sum_{i=1}^{3}(B_i k_y + C_i k_z)^2} \tag{13}$$

If we want to account for strain, we should expand to third order which gives an effective model:

$$\begin{aligned} H_3(k) = (E_0 + A_1 k_y^2 + A_2 k_z^2 + A_3 k_y k_z)I + (B_1 k_y + C_1 k_z + D_1 k_y^2 k_z + E_1 k_z^2 k_y)\sigma_x \\ +(B_2 k_y + C_2 k_z + D_2 k_y^2 k_z + E_2 k_z^2 k_y)\sigma_y + (B_3 k_y + C_3 k_z + D_3 k_y^2 k_z + E_3 k_z^2 k_y)\sigma_z \end{aligned} \tag{14}$$

With energy:

$$E_{3\pm}(k) = E_0 + A_1 k_y^2 + A_2 k_z^2 + A_3 k_y k_z \pm \sqrt{\sum_{i=3}^{3}(B_i k_y + C_i k_z + D_i k_y^2 k_z + E_i k_z^2 k_y)^2} \tag{15}$$

Note that our energy is symmetric under $(k_y, k_z) \to (-k_y, -k_z)$ even though the Hamiltonian is not. This explains the observed effective C_2 symmetry in the FS.

The free parameters are fit using simulated annealing. This gives the result shown in supplementary table I and II.

A_1	A_2	A_3	B_1	C_1	B_2	C_2	B_3	C_3
1.779	3.827	0.886	-0.248	-1.225	-0.138	-0.252	0.185	0.273

Supplementary Table I. Fit parameters for $E_{2\pm}$, see equation 13

A_1	A_2	A_3	B_1	C_1	D_1	E_1	B_2	C_2	D_2	E_2	B_3	C_3	D_3	E_3
1.895	8.051	2.701	0.145	0.021	-41.264	-150.345	0.082	-0.154	-2.524	-34.948	0.330	1.731	33.890	160.426

Supplementary Table II. Fit parameters for $E_{3\pm}$, see equation 15

SUPPLEMENTARY NOTE 3: DFT CALCULATION OF 2D CUTS

Although the triclinic structure of $RhBi_2$ only has inversion symmetry, $\mathbf{k} \to -\mathbf{k}$, when projected on the (100) surface, the inversion symmetry in 2D gives an effective twofold rotation symmetry. This can also be understood in the 2D cuts of the 3D FS as shown in Fig. 2

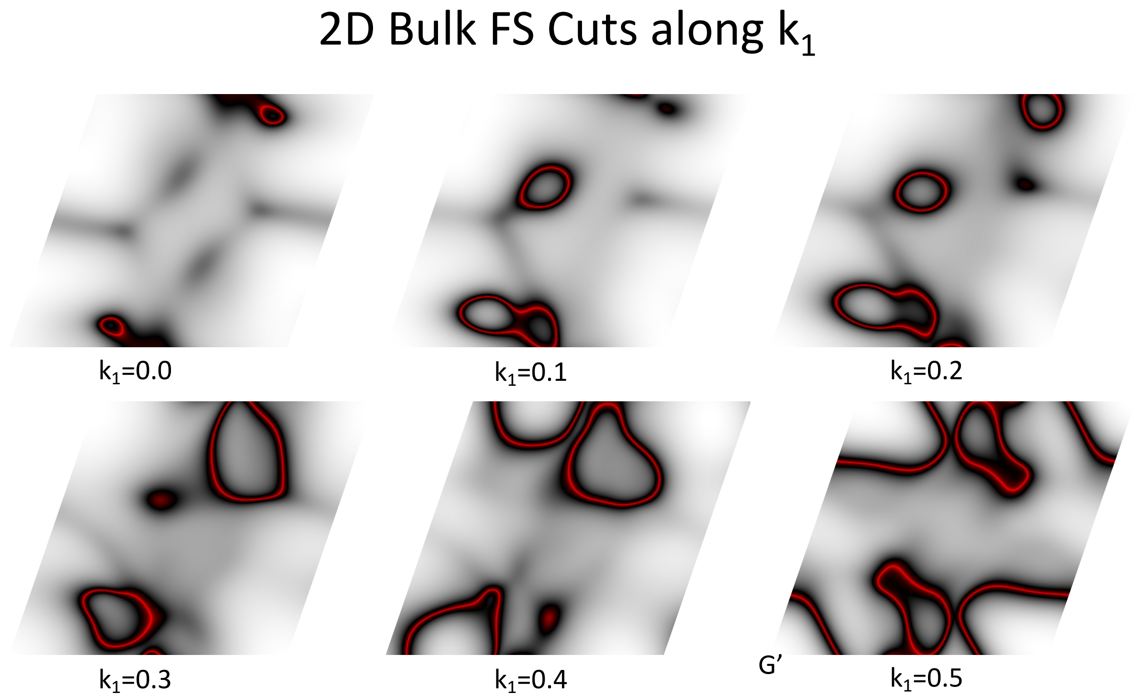

Supplementary Figure 2. **FS projection along $\mathbf{k}_1$ direction** 2D bulk Fermi surface cuts for $RhBi_2$ along k_1=0.0, 0.1, 0.2, 0.3, 0.4 and 0.5 planes. The 2-fold rotation symmetry only appears on the k_1=0.0 and 0.5 planes.

SUPPLEMENTARY NOTE 4: BAND STRUCTURE

A saddle point in the dispersion is characterized by a vanishing gradient, $\nabla \varepsilon_{\mathbf{k}} = 0$, and by opposite curvatures along orthogonal directions away from the extremum. Our ARPES measurements show evidence of the presence of a saddle point around the $\bar{\mathrm{Z}}$ point in the surface Brillouin zone (sBZ) of $RhBi_2$. To precisely determine the position of the saddle point, we perform a close investigation of the band structure using an effective low energy $k \cdot p$ model valid close to $\bar{\mathrm{Z}}$.

A first look into the band structure of the $k \cdot p$ model suggest the existence of two saddle points in the SBZ [see Fig.4 (a)]. Interestingly, as shown in Fig.3, the lower band $\varepsilon_{-,\mathbf{k}}$ is almost flat along the diagonal direction parametrized by

$$k_y = k_{\bar{\mathrm{Z}},y} + \alpha(k_z - k_{\bar{\mathrm{Z}},z}) \ , \tag{16}$$

where $\alpha \approx 4.15$ and $(k_{\bar{\mathrm{Z}},y}, k_{\bar{\mathrm{Z}},z}) \approx (0, 0.5)\frac{\pi}{a}$, denotes the position the $\bar{\mathrm{Z}}$ point. Zooming into the region around $\bar{\mathrm{Z}}$ clearly shows two local minima along this flat direction at $(k_y - k_{\bar{\mathrm{Z}},y}, k_z - k_{\bar{\mathrm{Z}},z}) = \pm(-0.042, 0.010)\frac{\pi}{a} \equiv \pm \mathbf{k}_0$. These points, on the other hand, behave as local maxima of the dispersion along the direction orthogonal to Eq. (16). This behavior is evidenced in Figs. 4(a) and (b), respectively.

In fact, the gradient of the lower band $\nabla \varepsilon_{-,\mathbf{k}}$ vanishes at $\pm \mathbf{k}_0$ [see Figs.4(c) and (d)], which makes these points true saddle points of the lower band. They occur at energy $E = -83$ meV, where the peak in the surface density of states occurs [see Fig.4 (a)].

Below, we provide details on the analytic solution of the gap equation (5) in the main text in the presence of a logarithmically divergent density of states $\rho(\xi) = \rho_0 \log(D/|\xi|)$. After the change of variables $y = \xi/T_c$, the gap equation can be rewritten as

$$1 = g\rho_0 \int_0^{\Lambda/T_c} dy \log\left(\frac{D/T_c}{y}\right) \frac{1}{y} \tanh\left(\frac{y}{2}\right) \ . \tag{17}$$

Using properties of the logarithm, this integral can be further divided into two contributions,

$$\frac{1}{g\rho_0} = \log\left(\frac{D}{T_c}\right) \int_0^{\Lambda/T_c} dy \frac{1}{y} \tanh\left(\frac{y}{2}\right) - \int_0^{\Lambda/T_c} dy \frac{\log(y)}{y} \tanh\left(\frac{y}{2}\right) \ . \tag{18}$$

The first integral in the right-hand-side of Eq.(18) is identical to the one encountered in the standard BCS gap equation when approximating the DoS by its (constant) value at the

Fermi level:

$$\int_0^{\Lambda/T_c} dy \frac{1}{y} \tanh\left(\frac{y}{2}\right) = \log\left(\frac{\kappa\Lambda}{T_c}\right) \ , \tag{19}$$

where $\kappa = 2e^{\gamma}/\pi \approx 1.13$, and γ is Euler's constant. For the second integral in Eq.(18), a simple integration by parts yields

$$\int_0^{\Lambda/T_c} dy \frac{\log(y)}{y} \tanh\left(\frac{y}{2}\right) = \frac{1}{2}\log^2\left(\frac{\Lambda}{T_c}\right) - \frac{1}{4}\int_0^{\Lambda/T_c} dy \log^2(y)\,\mathrm{sech}^2\left(\frac{y}{2}\right) \ . \tag{20}$$

Since $\Lambda/T_c \gg 1$ and $\log^2(y)\,\mathrm{sech}^2(y/2)$ remains finite as $y \to \infty$, we can approximate

$$\int_0^{\Lambda/T_c} dy \log^2(y)\,\mathrm{sech}^2\left(\frac{y}{2}\right) \approx \int_0^{\infty} dy \log^2(y)\,\mathrm{sech}^2\left(\frac{y}{2}\right) \approx \mathcal{C} \ , \tag{21}$$

where $\mathcal{C} = 2.669$ is a numerical constant. Substituting Eqs.(19) and (21) into Eq.(18), we obtain

$$\frac{1}{g\rho_0} = \log\left(\frac{D}{T_c}\right)\log\left(\frac{\kappa\Lambda}{T_c}\right) - \frac{1}{2}\log^2\left(\frac{\Lambda}{T_c}\right) + \frac{\mathcal{C}}{4} \ . \tag{22}$$

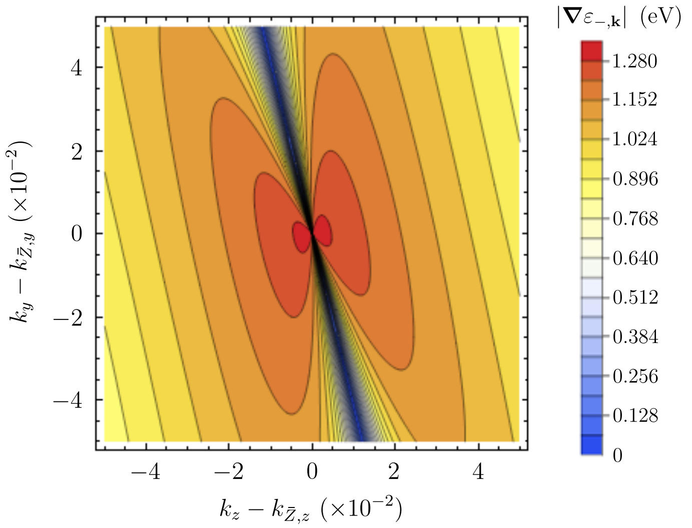

Supplementary Figure 3. Gradient of the lowest energy band of the $k \cdot p$ model. The direction where the norm of the gradient is minimum corresponds to the diagonal $k_y - k_{\bar{Z},y} = -4.15\,(k_z - k_{\bar{Z},z})$. In particular, it vanishes at $(k_{y0}, k_{z0}) = \pm(-0.042, 0.010)\frac{\pi}{a}$ with respect to $\bar{Z}$. These are saddle points of the lowest energy band. The momenta k_y and k_z are given in units of π/a. Accordingly, $|\nabla\varepsilon_{-,\mathbf{k}}|$ has dimensions of energy.

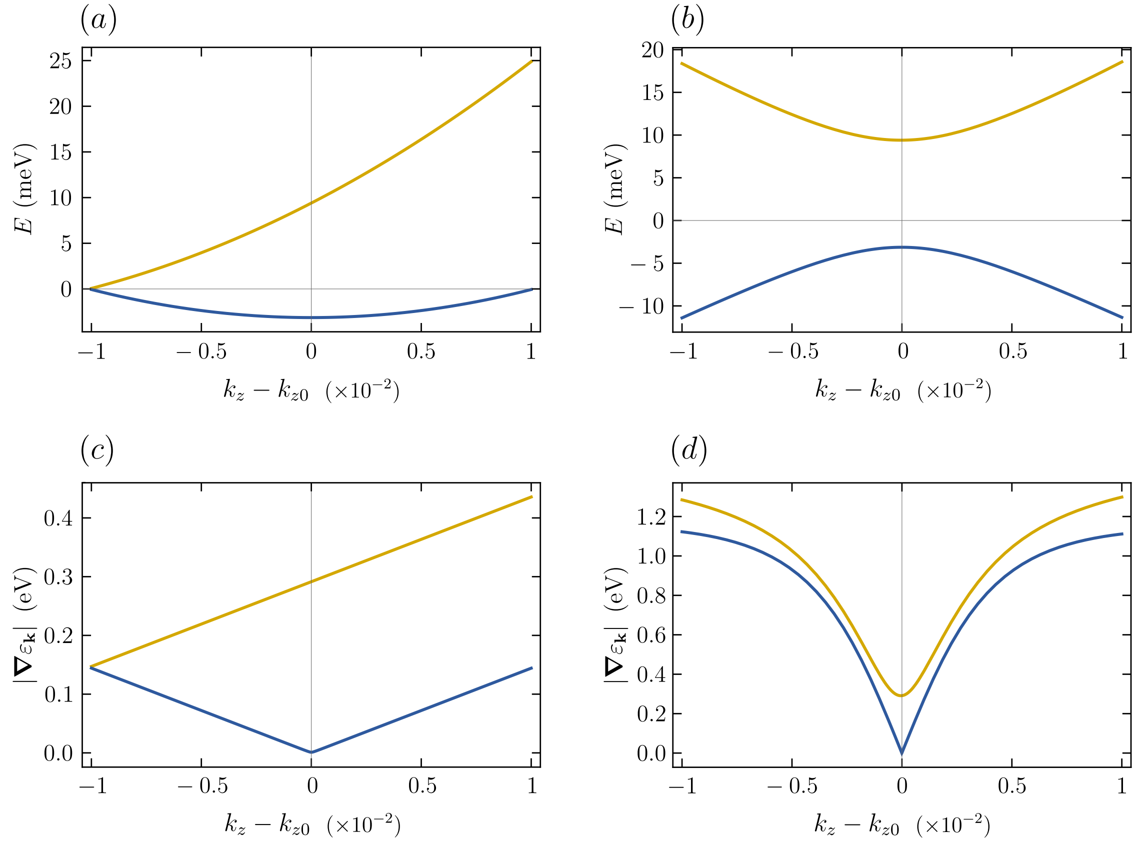

Supplementary Figure 4. Cuts of the band structure of the $k \cdot p$ model along the direction (a) $k_y - k_{\bar{Z},y} = -4.15\left(k_z - k_{\bar{Z},z}\right)$ and the orthogonal direction (b) $k_y - k_{\bar{Z},y} = 0.24\left(k_z - k_{\bar{Z},z}\right)$. Upper (lower) band is shown in yellow (blue). The dispersion is centered at the saddle point $\mathbf{k}_0 = (-0.042, 0.010)\frac{\pi}{a}$. Panels (c) and (d) show the norm of the gradient $|\nabla\varepsilon_{\mathbf{k}}|$ of the dispersion in **a** and **b**, respectively. The momenta k_y and k_z are given in units of π/a. Accordingly, $|\nabla\varepsilon_{\mathbf{k}}|$ has dimensions of energy.

Since typically we have $D > \Lambda^2$, it is convenient to rewrite the logarithms in Eq.(22) in terms of D/T_c rather than Λ/T_c. This simplifies Eq.(22) significantly, since the linear term in $\log(\kappa D/T_c)$ vanishes identically. We thus find

$$\frac{2}{g\rho_0} = \log^2\left(\frac{\kappa D}{T_c}\right) + \frac{\mathcal{C}}{2} - \log^2\left(\frac{\Lambda}{D}\right) - \log^2\left(\frac{1}{\kappa}\right) \,. \tag{23}$$

The strongest divergence comes from the term $\log^2(\kappa D/T_c)$, so that we can neglect the three last terms in the right-rand-side of Eq.(23), which yields T_c as given in Eq.(6) of the main text.

REFERENCES

[1] Liang Fu and Charles L Kane, "Topological insulators with inversion symmetry," Physical Review B **76**, 045302 (2007).

[2] J Labbe and J Bok, "Superconductivity in alcaline-earth-substituted La_2CuO_4: a theoretical model," EPL (Europhysics Letters) **3**, 1225 (1987).
